# Revolutionizing early HCC detection: groundbreaking validation of the HCC-check index for small tumors in Egyptian patients using CLIP and BCLC staging systems

**DOI:** 10.1007/s12032-025-02834-x

**Published:** 2025-06-19

**Authors:** Kareem A. Attallah, Khaled Farid, Mohamed S. Albannan

**Affiliations:** 1 Clinical Research Department, Damietta Directorate for Health Affairs, Egyptian Ministry of Health and Population, Damietta, Egypt; 2 Research and Development Department, Biotechnology Research Center, 23 July St., Industrial Zone, New Damietta, 34517 Egypt; 3 Faculty of Medicine, Mansoura University, Mansoura, Egypt

**Keywords:** BCLC, CLIP, HCC-Check, Liver, Nodule size

## Abstract

Hepatocellular carcinoma (HCC) is often associated with better treatment options for small asymptomatic tumors if HCC is caught early. A year ago, the “Technology in Cancer Research and Treatment” Journal published a report on the highly sensitive HCC-Check for the early diagnosis of HCC. Here, we examine and confirm the applicability of HCC-Check in 486 liver-diseased patients with earlier stages of HCC based on two widely used Cancer of the Liver Italian Program (CLIP) and Barcelona Clinic Liver Cancer (BCLC) staging systems for the identification of small tumors. Our results showed that HCC-Check produced 0.90 AUC with 78.26% sensitivity and 83.89% specificity, when used to distinguish small HCCs from cirrhotic patients. Our findings showed that HCC-Check increased with disease progression according to the CLIP and BCLC systems, producing significant (*p* < 0.0001) Spearman correlation coefficients of 0.633 and 0.458, respectively. HCC-Check yielded 0.85 (0.78–0.92) AUC, 66.67% (47.19–82.71) sensitivity, and 83.89% (77.69–88.94) specificity for identifying CLIP (0–1) while producing 0.87 (0.79–0.94) AUC, 65% (40.78–84.61) sensitivity, and 83.89% (77.69–88.94) specificity for identifying BCLC A. Additionally, HCC-Check yielded 0.90 (0.83–0.97) AUC, 78.26% (56.30–92.54) sensitivity, and 83.89% (77.69–88.94) specificity for discriminating small tumors < 3 cm. Simplified HCC-Check still competes with HCC-Check, providing 0.89 (0.83–0.95) AUC with 73.91% (51.60–89.77) sensitivity and 82.51% (76.22–87.72) specificity for identifying small-sized HCC. All these results were superior to those obtained by AFP. In conclusion, the study findings support that HCC-Check and its simplified version could be a useful tool for early detection of HCC, aiding definitive treatment.

## Introduction

Hepatocellular carcinoma (HCC) is a primary liver malignancy associated with significant morbidity and mortality [1]. Early detection of HCC is essential to improve patient outcomes, as treatment options are more effective in the early stages of the disease. However, current methods for detecting HCC are often invasive and not always reliable [2]. Based on the former issues, a cross-sectional study has recently been performed that yields the HCC-Check diagnostic test for early detection of HCC with a high degree of accuracy [3]. This index integrates serum levels of cytokeratin-1 (CK-1), epithelial membrane antigen (EMA), albumin, and alpha-fetoprotein (AFP) into a formula designed to maximize discrimination between HCC and non-malignant liver disease. The original study demonstrated high accuracy (AUC 0.95, 90% sensitivity, 84% specificity) for differentiating HCC from cirrhosis [3]. Its potential utility lies in providing a non-invasive, quantitative score that may enhance early HCC detection, particularly for smaller nodules or when AFP levels are non-diagnostic, thereby facilitating timely access to potentially curative treatments. Interestingly, HCC-Check values greater than 2.57 were found to increase the probability of occurrence of HCC with an estimated odds ratio of 50.65 indicating the higher susceptibility of patients to develop HCC than those with lower values. Additionally, HCC-Check allowed the correct identification of HCC patients with a small tumor less than 5 cm in size and a single nodule with AUCs of 0.92 and 0.85, respectively, indicating its ability to identify early HCC. While our previous work introduced the HCC-Check index [3], this study specifically validates its performance in the critical subgroup of patients with small hepatocellular carcinomas (< 3 cm) and those classified under early staging systems (CLIP 0–1 and BCLC A). This focus provides crucial evidence for the index’s utility in detecting HCC at its most treatable stages, an aspect not extensively detailed in the initial publication. Here, this work deals with the evaluation of HCC-Check in the early phases of the two widely used CLIP and BCLC scoring systems for smaller tumors [4, 5]. Indeed, these systems are widely accepted as reliable methods to evaluate the severity of HCC and determine treatment strategies [6]. By incorporating recent advances in imaging and laboratory tests [7], our index offers a non-invasive and accurate way of detecting and stage HCC at an earlier stage [3]. In this manuscript, the study is dedicated to introducing a simplified formula of HCC-Check without any numerical coefficients that can be easily applied and used by clinicians and then estimating its diagnostic performance against that of the original HCC-Check.

## Subjects and methods

### Samples

Patients with additional liver conditions or other suspected malignancies were not included in the current study. None of the HCC patients had received transarterial embolization, chemotherapy, radiofrequency ablation, or surgery. After being fully informed of the diagnostic procedures involved and the nature of the condition, each participant gave their informed written consent. The study protocol was in accordance with the ethical principles of the Declaration of Helsinki of 1975. The Research Ethics Committee of the Faculty of Pharmacy of Cairo University, Cairo, Egypt, gave its approval to the study protocol and informed consent (**BC 2912**).

The study included a total of 486 Egyptian patients, comprising 119 patients with HCC, 183 patients with liver cirrhosis (F4), and 184 patients with liver fibrosis (F1-F3). Within the HCC group, patients were staged according to CLIP (30 patients classified as CLIP 0–1, 24 as CLIP 2–3, 21 as CLIP 4) and BCLC (20 patients as BCLC A, 22 as BCLC B, 24 as BCLC C, 18 as BCLC D) systems. Exclusion criteria included co-infection with HBV, presence of other malignancies confirmed by biopsy, prior HCC-specific treatment (surgical resection, TACE, RFA, chemotherapy), and decompensated liver disease unrelated to HCC. A detailed patient selection flow diagram has been previously published [3].

On an automated biochemistry analyzer (A15, Biosystems, Spain), liver function tests were performed on fresh serum. Using the Immulite (1000) AFP kit, chemiluminescence was used to determine the AFP level (Diagnostic Products Corporation, Los Angeles, CA, USA). All patients had positive results for anti-HCV antibodies (Biomedica, Sorin, Italy) but negative results for HBsAg (Dia.Pro, Milan, Italy). Subsequently, the presence of HCV-RNA in the patients was verified using a quantitative polymerase chain reaction assay (COBAS Ampliprep/COBAS TaqMan, Roche Diagnostics, Pleasanton, USA). Subsequently, cytokeratin-1 (CK-1) and epithelial membrane antigen (EMA) were identified based on sodium dodecyl sulfate–polyacrylamide gel electrophoresis (SDS-PAGE) followed by Western blotting with a single immunoreactive band at 67 kDa and 130 kDa corresponding to CK-1 and EMA, respectively, as recently published [3]. They were then quantified in sera from patients based on an enzyme-linked immunosorbent assay (ELISA) [3].

The CLIP and BCLC staging systems were both used to define different stages of the HCC [4, 5]. BCLC staging approach, which is used in both clinical trials and everyday practice, has the support of both the American Association for the Study of Liver Diseases and the European Association for the Study of Liver [5]. In general, several clinical indicators, including the Child–Pugh score, tumor morphology, AFP level, and presence of portal vein thrombosis, are considered to stage patients according to the CLIP and BCLC scoring systems. HCC was established for patients with AFP levels of 400 U/L or higher through the detection of focal liver lesions by ultrasound (US). Subsequently, the diagnosis was confirmed by computed tomography and/or magnetic resonance imaging. Pathological confirmation was obtained where available/necessary. The early phases of HCC were described using CLIP (0–1) and BCLC (A). In addition to pathological information, the diagnosis of non-malignant chronic liver disease was based on the usual biochemical, clinical, and ultrasonographic standards. Liver fibrosis and cirrhosis were histopathologically classified according to FibroScan [7] (Echosens, Paris, France).

### Statistical analysis

Statistical Package for the Social Sciences (SPSS) software version 22.0 (SPSS Inc., Chicago, IL) software version 22.0 and GraphPad (GraphPad Software, San Diego, CA) Prism package version 5.0 were used to perform statistical analysis. The mean and standard deviation were used to express continuous variables. To correct for data deviance, the log transformation of the AFP values was carried out. A value of *p* < 0.05 was considered statistically significant. The represented formula was used to calculate the HCC-Check [3] index to differentiate HCC patients: HCC-Check: [2.879 + 0.009 × EMA (µg/ml)—0.236 × Albumin (g/dl) + 0.03 × CK-1 (µg/ml) + 0.299 × Log AFP (ng / ml)].

The simplified HCC-Check formula was developed primarily for enhanced clinical applicability and ease of calculation at the point of care, removing the need for specific coefficients as the following: ***Simplified HCC-Check* = [CK-1 (µg/ml) × Log AFP (ng/ml) × EMA (µg/ml) / Albumin (g/dL)].** The structure places established positive correlates of HCC (CK-1, AFP, EMA) in the numerator and a negative correlate (Albumin) in the denominator, based on findings from our original study [3] and known pathophysiology. While not derived from a separate regression analysis, this pragmatic simplification demonstrated competitive diagnostic performance compared to the original HCC-Check (AUC 0.92 vs 0.95, Table 1), supporting its potential utility.

**Table 1:** Diagnostic performances of Simplified HCC-Check for detecting HCC

Variables	Cut-off	AUC^*^ (95% CI)^►^, *p* value^**^	Diagnostic performances (%)		*X*^*2* $^; *p* value^**^
			Sensitivity (95% CI) ^►^	Specificity (95% CI) ^►^	
Liver fibrosis* VS* HCC ^♣^					
Simplified HCC-Check ^♯^	> 2.385	0.97 (0.95–0.98), < 0.0001	84.87 (77.15–90.78)	93.48 (88.88–96.59)	189.71, < 0.0001
Liver cirrhosis *VS* HCC ^♣^					
Simplified HCC-Check ^♯^	> 2.385	0.92 (0.89–0.95), < 0.0001	84.87 (77.15–90.78)	82.51 (76.22–87.72)	132.87, < 0.0001
Liver (Fibrosis + Cirrhosis) *VS* HCC ^♣^					
Simplified HCC-Check ^♯^	> 2.385	0.94 (0.92–0.97), < 0.0001	84.87 (77.15–90.78)	88.01 (84.24–91.15)	228.04, < 0.0001

^**♣**^*HCC* hepatocellular carcinoma, ^*^AUC area under the curve, ^**►**^*CI* confidence intervals, ^**$**^*X*^2^ qui square, ^●^*HCC-Check* [2.879 + 0.009 × EMA (µg/ml)—0.236 × Albumin (g/dL) + 0.03 × CK-1 (µg/ml) + 0.299 × LOG AFP (ng/ml)], ^**♯**^*Simplified HCC-Check* [CK-1/Albumin × LOG AFP (ng/ml) × EMA (µg/ml)]. ^**^p > 0.05 is considered non-significant, *p* < 0.05 is considered significant

The diagnostic effectiveness of HCC-Check and its simplified version was evaluated using AUC in relation to various stages and characteristics of the tumor. A 2 × 2 contingency table was used to calculate the sensitivity and specificity.

## Results

### Characteristics of the patient

A total of 486 Egyptian patients were classified into three groups: HCC = 119, liver cirrhosis (F4) = 183, and liver fibrosis (F1-F3) = 184, constituted this work. It should be noted that small HCC can be effectively cured by surgery with good clinical outcomes. Therefore, the main objective of this work was to detect and discriminate the early stages of patients with HCC with small nodules. In this study, patients who developed HCC produced a range of AFP values from normal (10 ng/ml) to more than 28,147 ng/ml. Normal levels of AFP are present in 29.4% (35 out of 119) of patients with HCC at the time of diagnosis and generally remain low, even with advanced HCC. An alpha-fetoprotein of 400 ng / ml is considered diagnostic for HCC; 27.7% (33 out of 119) of patients with HCC generated levels that are high. With values of this magnitude, the specificity of AFP is close to 99%. As expected, patients who acquired cirrhosis were younger than those who developed HCC, with a mean ± standard deviation age of 52.52 (± 8.97) years. Furthermore, patients who developed HCC were associated with a lower albumin level (2.91 ± 0.47) than cirrhotic patients (3.63 ± 0.63) with a significant difference (*p* < 0.0001). On the contrary, patients with HCC were accompanied by higher levels of aspartate aminotransferase (AST), alkaline phosphatase (ALP), and total bilirubin than those with cirrhosis with a significant difference (*p* < 0.0001). Furthermore, there were no significant differences between HCC and cirrhosis in terms of the mean value of alanine aminotransferase (ALT) and platelet count. Next, CK-1 and EMA, which were incorporated into the HCC-Check index ^3^, were identified and quantified in sera from patients according to Western blot and ELISA, respectively, as previously described. ^3^ As a result, HCC patients were associated with a higher concentration of CK-1 and EMA than cirrhosis patients with an extremely significant difference (*p* < 0.0001). In particular, the EMA concentration increased in HCC patients over F4 2.25 times. Unlike individuals who developed liver cirrhosis, patients with HCC showed a 10.79-fold increase in CK-1 levels.

### Application of HCC-check to distinguish different categories of HCC

As previously reported, the HCC-Check index was 90% sensitive and 84% specific, producing a superior AUC of 0.95 for discriminating HCC patients from cirrhotic ones. Here, a simplified version of HCC-Check was introduced, which is easier for doctors to use and utilize without any numerical coefficients and its diagnostic performance *versus* that of the original version was then compared. For simplicity, the positive correlation parameters (CK-1, EMA, and AFP) were placed in the numerator, while the negative correlation parameter (Albumin) was placed in the denominator to formulate the following function: Simplified HCC-Check: [CK-1 (µg/ml) × Log AFP (ng/ml) × EMA (µg/ml) /Albumin (g/dL)]. It is interesting to note that the simplified HCC-Check (AUC = 0.92) still competes with the original HCC-Check with a sensitivity of 84.87% and a specificity of 82.51% when a cut-off of 2.385 was applied to distinguish between participants with non-neoplastic liver cirrhosis and HCC patients, as provided in Table 1.

When confirming that our index can accurately diagnose tiny and early cancers, the size of the tumor was considered. To do this, patients with HCC were divided into patients with small tumors less than 3 cm in size and those with large tumors larger than or equal to 3 cm, as shown in Table 2. HCC-Check performed well in terms of diagnosing HCC characteristics in patients with a small tumor size < 3 cm, providing an AUC of 0.90 as summarized in Table 2. The subsequent step was concerned with the evaluation of HCC-Check and its simplified version in the early stages of the two widely used CLIP and BCLC staging systems for small tumors. HCC tumor stages were divided into [early (stages 0–1), intermediate (stage 2–3), and advanced (stage 4)] according to the CLIP staging system as shown in Table 3. Regarding the BCLC staging system, the HCC tumor stages were divided into [BCLC A ‘early,’ BCLC B ‘intermediate,’ BCLC C ‘advanced,’ and BCLC D ‘end’)] as shown in Table 4. Overall, our findings showed that HCC-check values increased with disease progression based on CLIP and BCLC scoring systems, producing significant (*p* < 0.0001) Spearman correlation coefficients of 0.633 and 0.458, respectively. Furthermore, HCC-Check has a good diagnostic capacity for identifying early stages of HCC, with performance reaching its peak in advanced stages. HCC-Check was found to produce an AUC of 0.85 (0.78–0.92), sensitivity of 66.67% (47.19–82.71), and a specificity of 83.89% (77.69–88.94) to identify CLIP (0–1) while producing an AUC of 0.87 (0.79–0.94), sensitivity of 65% (40.78–84.61), specificity of 83.89% (77.69–88.94) to identify BCLC A.

**Table 2:** Diagnostic performances of HCC-Check compared to its candidate markers for early detection of HCC according to tumor size

Categories	Cut-off	AUC^*^ (95% CI) ^►^, *p* value ^**^	Diagnostic performances (%)		*X*^*2* $^; *p* value ^**^
			Sensitivity (95% CI)^►^	Specificity (95% CI)^►^	
Liver cirrhosis *VS.* HCC ^♣^ [Nodule size < 3 cm]					
AFP (ng/ml) ^a^	> 400	0.72 (0.60–0.83), < 0.0001	21.74 (7.46–43.70)	99.46 (97.01–99.99)	32.45; < 0.0001
HCC-Check ^●^	> 2.57	0.90 (0.83–0.97), < 0.0001	78.26 (56.30–92.54)	83.89 (77.69–88.94)	44.274; < 0.0001
Simplified HCC-Check ^♯^	> 2.385	0.89 (0.83–0.95), < 0.0001	73.91 (51.60–89.77)	82.51 (76.22–87.72)	85.89; < 0.0001
Liver cirrhosis *VS.* HCC ^♣^ [Nodule size ≥ 3 cm]					
AFP (ng/ml) ^a^	> 400	0.82 (0.76–0.88), < 0.0001	31.76 (22.08–42.76)	99.46 (97.01–99.99)	60.46; < 0.0001
HCC-Check ^●^	> 2.57	0.96 (0.94–0.98), < 0.0001	91.67 (83.58–96.58)	83.89 (77.69–88.94)	139.954; < 0.0001
Simplified HCC-Check ^♯^	> 2.385	0.93 (0.90–0.96), < 0.0001	89.41 (80.85–95.04)	81.97 (75.62–87.25)	124.80; < 0.0001

^**a**^*AFP* alpha-fetoprotein, ^**b**^*CK-1* cytokerain-1, ^**c**^*EMA* epithelial membrane antigen, ^**♣**^*HCC* hepatocellular carcinoma, ^*^AUC area under the curve, ^**►**^*CI* confidence intervals, ^**$**^*X*^2^ qui square, ^●^*HCC-Check*: [2.879 + 0.009 × EMA (µg/ml)—0.236 × Albumin (g/dL) + 0.03 × CK-1 (µg/ml) + 0.299 × LOG AFP (ng/ml)], ^**♯**^*Simplified HCC-Check* [CK-1/Albumin × LOG AFP (ng/ml) × EMA (µg/ml)]. ^**^p > 0.05 is considered non-significant; *p* < 0.05 is considered significant

**Table 3:** Diagnostic performances of HCC-Check and Simplified HCC-Check compared to its candidate markers for early detection of HCC according to CLIP scoring system

Categories	Cut-off	AUC^*^ (95% CI) ^►^, *P* value ^**^	Diagnostic performances (%)		*X*^*2* $^; *p* value ^**^
			Sensitivity (95% CI) ^►^	Specificity (95% CI) ^►^	
Liver cirrhosis* VS.* HCC ^♣^ [CLIP ^Ꞵ^ 0–1 (early)]					
AFP (ng/ml) ^a^	> 400	0.66 (0.54–0.77), 0.006	16.67 (56.42–34.72)	99.46 (97.01–99.99)	24.47; < 0.0001
HCC-Check ^●^	> 2.57	0.85 (0.78–0.92), < 0.0001	66.67 (47.19–82.71)	83.89 (77.69–88.94)	36.739; < 0.0001
Simplified HCC-Check ^♯^	> 2.385	0.85 (0.78–0.91), < 0.0001	66.67 (47.19–82.71)	82.51 (76.22–87.72)	33.78; < 0.0001
Liver cirrhosis* VS.* HCC ^♣^ [CLIP ^Ꞵ^ 2–3 (intermediate)]					
AFP (ng/ml) ^a^	> 400	0.86 (0.77–0.95), < 0.0001	29.17 (12.62–51.09)	99.46 (97.01–99.99)	46.78; < 0.0001
HCC-Check ^●^	> 2.57	0.98 (0*.*96–1.00), < 0.0001	100.0 (85.75–100.0)	83.89 (77.69–88.94)	77.494; < 0.0001
Simplified HCC-Check ^♯^	> 2.385	0.93 (0.88–0.98), < 0.0001	91.67 (73.00–98.97)	81.97 (75.62–87.25)	60.55; < 0.0001
Liver cirrhosis* VS.* HCC ^♣^ [CLIP ^Ꞵ^ ≥ 4 (advanced)]					
AFP (ng/ml) ^a^	> 400	0.92 (0.84–1.01), < 0.0001	76.19 (52.83–91.78)	99.46 (97.01–99.99)	141.11; < 0.0001
HCC-Check ^●^	> 2.57	0.9997 (0.99–1.001), < 0.0001	100.0 (83.16–100.0)	83.89 (77.69–88.94)	68.481; < 0.0001
Simplified HCC-Check ^♯^	> 2.385	0.97 (0.93–1.006), < 0.0001	95.24 (76.18–99.88)	81.97 (75.62–87.25)	59.96; < 0.0001

^**a**^*AFP* alpha-fetoprotein, ^**♣**^*HCC* hepatocellular carcinoma, ^*^AUC area under the curve; ^**►**^*CI* confidence intervals, ^**$**^*X*^2^ qui square, ^**Ꞵ**^*CLIP* Cancer of the Liver Italian Program staging, ^●^*HCC-Check* [2.879 + 0.009 × Epithelial membrane antigen (µg/ml)—0.236 × Albumin (g/dL) + 0.03 × Cytokerain-1 (µg/ml) + 0.299 × LOG AFP (ng/ml)], ^**♯**^Simplified HCC-Check: [Cytokerain-1/Albumin × LOG AFP (ng/ml) × Epithelial membrane antigen (µg/ml)]. ^**^p > 0.05 is considered non-significant; *p* < 0.05 is considered significant

**Table 4:** Diagnostic performances of HCC-Check and Simplified HCC-Check compared to its candidate markers for early detection of HCC according to BCLC scoring system

Categories	Cut-off	AUC ^*^ (95% CI) ^►^, *P* value ^**^	Diagnostic performances (%)		*X*^*2* $^; *p* value ^**^
			Sensitivity (95% CI) ^►^	Specificity (95% CI) ^►^	
Liver cirrhosis* VS.* HCC ^♣^ [BCLC ^ȸ^ A (early)]					
AFP (ng/ml) ^a^	≥ 400	0.66 (52.58–78.76), 0.02	10.00 (1.24–31.70)	99.46 (97.01–99.99)	11.07; < 0.001
HCC-Check ^●^	> 2.57	0.87 (0.79–0.94), < 0.0001	65.00 (40.78–84.61)	83.89 (77.69–88.94)	25.933; < 0.0001
Simplified HCC-Check ^♯^	> 2.385	0.85 (0.77–0.93), < 0.0001	65.00 (40.78–84.61)	81.97 (75.62–87.25)	23.59; < 0.0001
Liver cirrhosis* VS.* HCC ^♣^ [BCLC ^ȸ^ B (intermediate)]					
AFP (ng/ml) ^a^	≥ 400	0.80 (67.21–92.03), < 0.0001	45.45 (24.39–67.79)	99.46 (97.01–99.99)	77.99; < 0.0001
HCC-Check ^●^	> 2.57	0.94 (0.88–0.99), < 0.0001	86.36 (65.09–97.09)	83.89 (77.69–88.94)	59.695; < 0.0001
Simplified HCC-Check ^♯^	> 2.385	0.90 (0.83–0.96), < 0.0001	86.36 (65.09–97.09)	81.97 (75.62–87.25)	49.85; < 0.0001
Liver cirrhosis* VS.* HCC ^♣^ [BCLC ^ȸ^ C (advanced)]					
AFP (ng/ml) ^a^	≥ 400	0.86 (76.49–95.53), < 0.0001	37.50 (18.80–59.41)	99.46 (97.01–99.99)	63.02; < 0.0001
HCC-Check ^●^	> 2.57	0.96 (0.92–1.00), < 0.0001	95.65 (78.05–99.89)	83.89 (77.69–88.94)	68.591; < 0.0001
Simplified HCC-Check ^♯^	> 2.385	0.93 (0.89–0.98), < 0.0001	87.50 (67.64–97.34)	81.97 (75.62–87.25)	54.60; < 0.0001
Liver cirrhosis* VS.* HCC ^♣^ [BCLC ^ȸ^ D (end-stage)]					
AFP (ng/ml) ^a^	≥ 400	0.90 (80.75–0.99), < 0.0001	44.44 (21.53–69.24)	99.46 (97.01–99.99)	73.84; < 0.0001
HCC-Check ^●^	> 2.57	0.996 (0.98–1.004), < 0.0001	100 (81.47–100.00)	83.89 (77.69–88.94)	63.613; < 0.0001
Simplified HCC-Check ^♯^	> 2.385	0.99 (0.98–1.002), < 0.0001	100 (81.47–100.00)	81.97 (75.62–87.25)	59.71; < 0.0001

^**a**^*AFP* alpha-fetoprotein, ^**♣**^*HCC* hepatocellular carcinoma, ^*^AUC area under the curve, ^**►**^*CI* confidence intervals, ^**$**^*X*^2^ qui square, ^**ȸ**^*BCLC* Barcelona clinic liver cancer staging, ^●^*HCC-Check* [2.879 + 0.009 × Epithelial membrane antigen (µg/ml)—0.236 × Albumin (g/dL) + 0.03 × Cytokerain-1 (µg/ml) + 0.299 × LOG AFP (ng/ml)], ^**♯**^*Simplified HCC-Check* [Cytokerain-1/Albumin × LOG AFP (ng/ml) × Epithelial membrane antigen (µg/ml)]. ^**^p > 0.05 is considered non-significant; *p* < 0.05 is considered significant

The receiver operating characteristic (ROC) analysis further demonstrated that HCC-Check and its simplified formula outperformed serum AFP in distinguishing early patients with HCC. These findings support previous findings that AFP is not responsive to small HCCs [8].

## Discussion

Recently, there has been a growing interest in applying indexes in the early detection of many types of cancer, particularly HCC. This could be explained by the fact that only patients whose tumors are detected early can access effective treatments that have the best chance of reversing HCC [9]. Indeed, surgical excision offers a promising prognosis if detected early with 5-year survival rates of more than 70% [10]. However, the poor prognosis and high mortality of HCC are attributed to the absence of surveillance and the lack of early diagnostic precision. Globally, imaging methods and biomarkers are considered the main HCC screening strategies [11]. Ultrasound (US) imaging technique is used most frequently in HCC screening because it is simple, non-invasive, affordable, and enables real-time monitoring. However, determining the actual sensitivity of the US is difficult and depends on the available equipment, the texture of the liver echo, and the doctor’s skill [12]. Without doubt, the most common biomarker for the identification of HCC is AFP and its glycated isoforms continue to be ineffective as the exclusive method of detecting HCCs in their early stages [13]. Therefore, the development of a reliable and efficient method for early detection of HCC would be crucial to improving the prognosis of the condition and increasing the proportion of patients eligible for curative treatment [14].

Practically, the type of tumor, its size, the extent of cancer dissemination, the health of the liver, and the time since the cancer was discovered all affect the course of treatment and prognosis for HCC. More recently, a new index ‘HCC-Check diagnostic test’ has been developed for the early detection of HCC with a high degree of precision [3]. It is noteworthy that HCC-Check enabled accurate detection of HCC, providing superior AUCs of 0.99 and 0.95 in patients with liver fibrosis and cirrhosis, respectively. The diagnostic capability of HCC-Check for distinguishing different categories of HCC has already been investigated. The results showed that the HCC-Check produced competitive AUCs of 0.92 and 0.98 for predicting HCC patients who have single and multiple nodules of cirrhotic ones, respectively. Additionally, the HCC-Check gave higher AUC of 0.98 and 0.93 for discriminating HCC patients with and without portal vein thrombosis from those with cirrhosis, respectively.

It should be noted that our developed index has previously succeeded in accurate detection of small nodules less than 5 cm in size, giving 0.85 AUC. Here, our challenge was to make worthy improvements in this index reliability and its simplified formula by verifying its use in diagnosing small nodules less than 5 cm in size but also less than 3 cm in size, which in turn support the efficacy of HCC-Check in early detection of HCC. By confirming that our index can accurately diagnose tiny and early cancers, we have achieved considerable improvements in its dependability. Surprisingly, AUCs of 0.90 and 0.89 were obtained for both HCC-Check and its simplified version for detecting small nodules less than 3 cm in size, respectively. These findings were promising in reducing the poor prognosis of HCC and thus improving more treatment options. We showed that large tumors (≥ 3 cm) were significantly (*p* < 0.0001) higher HCC-Check values.

The application of different staging systems is crucial both for management planning and for collecting prognostic information. Many different staging strategies have been suggested for HCC [15]. CLIP and BCLC staging methods are the ones that are used most frequently for HCC classification. We showed that the advanced stages of the two widely used staging methods, CLIP and BCLC, were significantly (*p* < 0.0001) higher HCC-Check values. HCC staging systems take seriously both the provision of prognostic information and the design of management [13]. The high diagnostic capacity of HCC-Check to detect HCC in early CLIP (0–1; AUC = 0.85, sensitivity = 66.67%) tumor stage and early BCLC (BCLC A; AUC = 0.87, sensitivity = 65%) is another attractive feature. Based on these findings, it could be concluded that HCC-Check and its condensed version outperform AFP by having higher AUCs for HCC detection from non-malignant liver cirrhosis and the ability to correctly identify HCC patients with tumor sizes < 3 cm, CLIP (0–1) and BCLC (A). The sensitivity of the HCC-Check and its condensed version was higher than that of other HCC diagnostic methods, such as ultrasound, which has a sensitivity between 60 and 80%, CT, which has a sensitivity of 72%, and MRI, which has a sensitivity of 79% [16]. Furthermore, the results of this study compared favorably with those of other markers utilized in the early diagnosis of HCC. For example, AFP-L3 has a sensitivity of just 35% in individuals with small HCCs [17]. Only 15–30% of patients with early stages of HCC have elevated levels of decarboxyprothrombin circulation, compared to 50–60% of all patients [18].

Regarding cost-effectiveness, the HCC-Check index incorporates markers (CK-1, EMA) that may not be part of routine liver disease panels, potentially increasing upfront laboratory costs compared to AFP testing alone. However, the potential economic benefits should also be considered. By improving the detection rate of early-stage, curable HCC, the index could lead to significant downstream savings by reducing the need for expensive late-stage treatments and improving patient outcomes. Furthermore, if proven sufficiently accurate, it might potentially optimize the use of more expensive imaging resources in surveillance protocols. A formal cost-effectiveness analysis comparing HCC-Check-based strategies with current standards is warranted but was beyond the scope of this study.

This study has several limitations that should be acknowledged. Firstly, the findings are based on a single-center cohort of Egyptian patients, and external validation in geographically and ethnically diverse populations is required to assess the generalizability of the HCC-Check index. Secondly, the cross-sectional design limits our ability to evaluate the index’s prognostic significance or its direct impact on clinical management and long-term outcomes, such as survival rates; prospective longitudinal studies are necessary for this purpose. Thirdly, while we demonstrated HCC-Check’s superiority over serum AFP, direct comparative analyses against the performance of standard imaging modalities (ultrasound, CT, MRI) or other relevant biomarkers like AFP-L3 and DCP were not conducted within this specific study cohort, primarily due to limitations in routine clinical availability for the latter. Such comparisons are important avenues for future research. Additionally, while the simplified HCC-Check formula demonstrated competitive diagnostic performance, its derivation was based on pragmatic considerations for clinical utility rather than separate statistical modeling. Finally, a formal cost-effectiveness analysis comparing surveillance strategies incorporating the HCC-Check index against current standards was beyond the scope of this study and warrants future investigation.

## Conclusion

The study findings support the idea that HCC-Check and its condensed version could be used as a useful tool for early detection of HCC, which may help with definitive treatment.

## Data Availability

Data is included in this manuscript.

## Abbreviations

AFP: Alpha fetoprotein
ALP: Alkaline phosphatase
ALT: Alanine aminotransferase
AST: Aspartate aminotransferase
AUC: Area under curve
BCLC: Barcelona Clinic Liver Cancer
CLIP: Cancer of the Liver Italian Program
CK-1: Cytokeratin-1
ELISA: Enzyme-linked immunosorbent assay
EMA: Epithelial membrane antigen
HCC: Hepatocellular carcinoma
ROC: Receiver operating characteristic
SDS-PAGE: Sodium dodecyl sulfate–polyacrylamide gel electrophoresis
US: Ultrasound
